# Neuropsychological and neuroimaging preliminary results supporting prodromal dementia with Lewy bodies criteria

**DOI:** 10.1002/alz70857_102865

**Published:** 2025-12-25

**Authors:** Giulia Zazzaro, Desirée Conti, Massimiliano Panigutti, Giulia Bechi Gabrielli, Marco Serrentino, Micaela Sepe‐Monti, Giuseppina Talarico, Marco Canevelli, Giuseppe Bruno, Fabrizia D'Antonio

**Affiliations:** ^1^ Fondazione Santa Lucia IRCCS, Rome, Italy; ^2^ Sapienza University of Rome, Rome, Italy; ^3^ Sapienza University of Rome, Rome, Rome, Italy

## Abstract

**Background:**

The prodromal stage of dementia with Lewy bodies (pro‐DLB) has been recently defined [1]; however, the neuropsychological and neuroimaging profile of these patients according to the possible clinical onset (i.e., mild cognitive impairment [MCI], psychiatric‐onset, delirum‐onset) has not been thoroughly investigated yet. In this study, we aimed to clarify the neuropsychological and neuroimaging markers of pro‐DLB.

**Method:**

We included 26 patients with pro‐DLB (17 MCI‐DLB, 8 psychiatric‐onset, 1 delirium‐onset) and 15 with MCI due to Alzheimer's disease (AD). All patients underwent an extensive neuropsychological battery assessing all cognitive domains and neuropsychiatric symptoms. Qualitative neuropsychological indices were developed to capture subtle differences in pro‐DLB. All 26 pro‐DLB patients, a subgroup of 12 MCI‐AD patients, and 13 healthy controls also underwent a structural MRI acquisition. Voxel‐based Morphometry (VBM) analyses were performed to compare differences in gray matter volume between the groups.

**Result:**

In comparison to MCI‐AD, pro‐DLB patients showed a worse performance in computerized tests of alertness and working memory, in visuo‐perceptive, visuo‐spatial and visuo‐constructive processing, in abstract reasoning and planning tests. While, the same outcomes were observed when comparing MCI‐DLB with MCI‐AD, psychiatric pro‐DLB patients obtained lower scores than MCI‐AD only on computerized trials of alertness. Regarding neuropsychiatric symptoms, pro‐DLB patients had greater anxiety and more severe and frequent hallucinations. Moreover, pro‐DLB patients showed increased atrophy in occipital areas, mainly in early and ventral visual systems, in the inferior parietal lobule, and in frontal regions (e.g., anterior cingulate and orbitofrontal cortex, especially in psychiatric pro‐DLB). Subcortically, atrophy was found in the nucleus accumbens. Relative preservation of the medial temporal lobe was found in pro‐DLB relative to MCI‐AD.

**Conclusion:**

In line with the full‐blown disease, the neuropsychological profile of pro‐DLB patients is characterized by deficits in visuo‐perceptive, visuo‐spatial and visuo‐constructive abilities as well as executive functions and attention. Accordingly, the neuropsychological assessment should focus on the evaluation of these cognitive domains employing the most sensitive tests (including computerized ones). Notably, the localization of brain atrophy is coherent not only with neuropsychological deficits and neuropsychiatric symptoms, but also with the different phenotypes of pro‐DLB.

